# Experimental Infection of Rabbits with Rabbit and Genotypes 1 and 4 Hepatitis E Viruses

**DOI:** 10.1371/journal.pone.0009160

**Published:** 2010-02-11

**Authors:** Hongxia Ma, Lin Zheng, Yunbo Liu, Chenyan Zhao, Tim J. Harrison, Yuyuan Ma, Shuhua Sun, Jingang Zhang, Youchun Wang

**Affiliations:** 1 Department of Cellular Biology, National Institute for the Control of Pharmaceutical and Biological Products, Beijing, China; 2 Laboratory for Viral Detection, National Center of Biomedical Analysis at the Institute of Transfusion Medicine, AMMS, Beijing, China; 3 Institute of Laboratory Animal Science, Chinese Academy of Medical Sciences and Peking Union Medical College, Panjiayuan, Beijing, China; 4 Henan Center for Disease Control and Prevention, Zhengzhou, China; 5 Division of Medicine, University College London Medical School, Windeyer Building, London, United Kingdom; Saint Louis University School of Medicine, United States of America

## Abstract

**Background:**

A recent study provided evidence that farmed rabbits in China harbor a novel hepatitis E virus (HEV) genotype. Although the rabbit HEV isolate had 77–79% nucleotide identity to the mammalian HEV genotypes 1 to 4, their genomic organization is very similar. Since rabbits are used widely experimentally, including as models of infection, we investigated whether they constitute an appropriate animal model for human HEV infection.

**Methods:**

Forty-two SPF rabbits were divided randomly into eleven groups and inoculated with six different isolates of rabbit HEV, two different doses of a second-passage rabbit HEV, and with genotype 1 and 4 HEV. Sera and feces were collected weekly after inoculation. HEV antigen, RNA, antibody and alanine aminotransferase in sera and HEV RNA in feces were detected. The liver samples were collected during necropsy subject to histopathological examination.

**Findings:**

Rabbits inoculated with rabbit HEV became infected with HEV, with viremia, fecal virus shedding and high serum levels of viral antigens, and developed hepatitis, with elevation of the liver enzyme, ALT. The severity of disease corresponded to the infectious dose (genome equivalents), with the most severe hepatic disease caused by strain GDC54-18. However, only two of nine rabbits infected with HEV genotype 4, and none infected with genotype 1, developed hepatitis although six of nine rabbits inoculated with the genotype 1 HEV and in all rabbits inoculated with the genotype 4 HEV seroconverted to be positive for anti-HEV IgG antibody by 14 weeks post-inoculation.

**Conclusions:**

These data indicate that rabbits are an appropriate model for rabbit HEV infection but are not likely to be useful for the study of human HEV. The rabbit HEV infection of rabbits may provide an appropriate parallel animal model to study HEV pathogenesis.

## Introduction

Hepatitis E virus (HEV) is transmitted between humans by the fecal-oral route and causes an acute, self-limiting hepatitis with high morbidity in young adults. Hepatitis E is an important public health concern in many developing countries in Asia and Africa and occurs sporadically in some industrialized countries. HEV is a small, non-enveloped virus with a single-stranded, positive-sense RNA genome of approximately 7.2 kb containing three open reading frames (ORFs), ORF1, ORF2 and ORF3, where ORF3 partially overlaps ORF2 [1], [2], [3]. Currently, there are four recognized genotypes of HEV that can infect mammals but only one serotype [4], [2], [5], [6], [7], [8].

Since the first non-human strain of HEV was isolated from a pig in the United States in 1997 and designated swine HEV [9], antibodies to HEV have been detected in a wide range of domestic and wild mammals, including cattle, sheep, goats, horses, rats and rabbits [10], [11], [12]. Zoonotic transmission of HEV is increasingly recognized and virological evidence of infection with HEV has been found in domestic swine, wild boar, deer and horses, particularly genotypes 3 and 4 [13], [12], [14]. Avian HEV was characterized recently in chickens and is genetically and antigenically related to the mammalian genotypes but is phylogenetically distinct [15]. A recent study provided evidence that farmed rabbits in China harbor a reservoir of a novel genotype of HEV [10]. Although the overall nucleotide similarity of rabbit HEV is only 77–79%, compared with genotypes 1–4, the relationship of rabbit HEV to the mammalian genotypes is much closer than to avian HEV.

As the presumed natural reservoir of HEV genotypes 3 and 4, pigs are able to amplify the virus but only develop subclinical disease [16], [13], [17]. Although pigs could be used as an experimental model of genotypes 3 and 4 HEV infection but cannot be infected with genotype 1 HEV and difficulties in handling, manipulating, and housing [18], [17], [19] and above all, the inability to emulate human disease caused by genotypes 3 and 4, limit the value of this animal model for studying the disease aspect of HEV infection. A number of non-human primate species, such as cynomolgus macaques, rhesus, owl, and African green monkeys and chimpanzees are susceptible to HEV infection and some have been used successfully as animal models of hepatitis E [20], [21], [22], [23], [24], [25]. The most valuable are macaques because the disease is reproduced readily in them. The course of infection in experimentally-infected primates is similar to that in humans with some variations in incubation periods [20], [21], [22], [23], [24], [25]. However, these non-human primate animal models also are restricted by limited resources, ethical concerns, and difficult and expensive experimental procedures. Another variety of HEV was identified and characterized in chickens and evaluated as a model for the study of HEV infection [26]. Although the genomic organization of avian HEV is very similar to HEV genotypes 1–4, the overall nucleotide identity is only around 50% [27] and the virus is phylogenetically distinct from the mammalian HEV genotypes [15].

At present, the advancement of HEV research has been hampered to by the lack of an effective *in vitro* culture system and a small animal model of HEV infection would be extremely valuable. This study investigated the experimental infection of specific pathogen-free (SPF) rabbits with HEV isolated from rabbits, to study systematically HEV pathogenesis and replication of rabbit HEV in its natural host. Furthermore, we also studied the infection of rabbits with HEV genotypes 1 and 4 to characterize the clinical course associated with cross-species HEV infection.

## Materials and Methods

### Ethics Statement

The animal experimentation was approved by the Committee of Laboratory Animal Welfare and Ethics, National Institute for the Control of Pharmaceutical and Biological Products. The regulation for the review committee of laboratory animal welfare and ethics and protocol for the review on laboratory animal welfare and ethics, National Institute for the Control of Pharmaceutical and Biological Products, were followed.

### Viruses

The rabbit strain of HEV was originally recovered from serum samples from rabbits bred in two farms in Gansu province, China [10]. All six of the original rabbit HEV infectious plasma samples and the second-passage samples (GDC54-18) of rabbit HEV were generated from rabbits and titered by real-time PCR. The titers of GDC9, RC12, GDC22, GDC37, GDC46, GDC54 and GDC54-18 used as the inocula for this study, were 1.03×10^3^, 4.69×10^1^, 6.35×10^3^, 2.59×10^4^, 6.40×10^4^, 6.93×10^4^ and 6.74×10^6^ genome equivalents per mL, respectively. The second-generation infectious stock of GDC54-18 was recovered from the feces of a rabbit experimentally-infected with the GDC54 HEV strain during the acute phase of infection. The virus suspensions were stored at −70°C for later use. The HEV RNA content of virus samples were determined prior to inoculation by reverse transcription-polymerase chain reaction (RT-PCR), as described previously [20], [23].

Two strains of HEV isolated from humans were used for challenge. A genotype 4 strain (RH4) was collected from the feces of an individual with acute hepatitis E in Beijing, China and a genotype 1 strain (RH1) was collected from the feces of patients with sporadic hepatitis E in the Xinjiang Autonomous Region, China [20], [28], [29]. The fecal samples were diluted in phosphate-buffered saline (PBS; pH 7.4) containing 1% bovine serum albumin (BSA) to make a 10% (wt/vol) suspension, clarified by centrifugation at 3000 rpm at room temperature for 10 min and filtered through 0.45 µm and 0.22 µm filters. The resulting suspensions were aliquotted at 1 ml/vial and kept under liquid nitrogen for later analysis. Rabbits were inoculated with 1.10×10^6^ genome equivalents of RH1 or 1.14×10^7^ genome equivalents of RH4 [30].

### Animals

Forty-two 7-week old SPF rabbits weighing between 800 and 1000 g were obtained from the National Institute for the Control of Pharmaceutical and Biological Products, in Beijing, China. Prior to inoculation, all rabbits were confirmed negative for anti-rabbit HEV antibodies and antigens by an enzyme-linked immunosorbent assay (ELISA).

### Experimental Design

Rabbits were divided randomly into eleven groups and inoculated intravenously with different doses of the various HEV isolates (Table 1). Each rabbit was housed in a separate cage and fed twice a day, with access to drinking water *ad libitum*.

**Table 1:** Challenge schedules for rabbits inoculated with different HEV strains.

Inoculum strain (Groups)	Animals per group	Infectious dose: genome equivalents
RH1	9	1.10×10^6^
RH4	9	1.14×10^7^
GDC9	2	1.03×10^3^
RC12	2	4.69×10^1^
GDC22	2	6.35×10^3^
GDC37	2	2.59×10^4^
GDC46	2	6.40×10^4^
GDC54	2	6.93×10^4^
Control	4	0
1GDC54-18	4	6.74×10^6^
2GDC54-18	4	1.35×10^7^

### Sample Collection and Processing

Blood samples and feces were collected weekly following virus challenge. Serum samples were tested for levels of the liver enzyme, alanine aminotransferase (ALT), HEV antigen and anti-HEV antibodies using standard methods, as described below. Serum and feces samples were tested weekly for HEV RNA by real-time PCR, as reported previously [31]. Furthermore, positive samples were tested using reverse transcription-PCR (RT-PCR) with another two pairs of primers [20], [23], [28] and the positive amplicons sequenced. In fact, there was no animal necropsied at planned time because the number of rabbits for each group was limited. Thus only a few rabbits were necropsied randomly following accidental death during the study or at the end of the study, and samples of liver were collected.

### Pathology and Histopathology

No gross pathological lesions were observed in the livers during necropsies. Liver samples collected at necropsy were fixed in 10% neutral buffered formalin and processed for routine histological examination. Each specimen was embedded in paraffin and cut into 3 µm serial sections, stained with hematoxylin–eosin and subjected to histopathological examination by light microscopy.

### Determination of ALT Concentrations

All rabbits were monitored weekly after inoculation for 14 weeks. ALT concentrations in sera were measured on the day of collection using an automated analyzer (Hitachi 912; Roche, Indianapolis, USA) according to the manufacturer's instructions. Biochemical evidence of hepatitis was recorded when the serum ALT concentration exceeded the baseline ALT level by more than two-fold, as defined by a peak ALT value that was equal to or greater than twice the pre-challenge values. These criteria were based on the correlation between the level of ALT increase and liver pathology described in a previous study using a non-human primate as an animal model of hepatitis [23].

### ELISA for HEV Antigen and Antibody

All serum samples were tested for the presence of HEV antigen as described previously [32] and for antibody by an enzyme-linked immunosorbent assay based on the virus E2 protein (amino acids 394–606 of HEV ORF2) [23], according to the manufacturer's instructions (Wantai, Beijing, China). Sample/cutoff (S/CO) values were calculated and values >1 were considered positive, as determined previously [29]. Convalescent-phase sera from experimentally-infected rabbits and sera from unbinfected SPF rabbits were included as positive and negative controls, respectively.

### RT-PCR to Detect HEV RNA

To determine the genomic dose of each inoculum, RNA was extracted from sera and fecal suspensions using a spin-column kit and the procedures of Kinghawk Biopharmaceutical Company (Beijing, China) and evaluated by a real-time fluorescence RT-PCR assay with primers based on the conserved regions of HEV ORF3, as reported previously [31]. In order to detect HEV RNA in the feces and sera of inoculated and non-inoculated rabbits, RNA was extracted from those samples using the method described above and tested for HEV genomic RNA by nested RT-PCR with primers derived from the ORF2 region [20], [23], [28]. A final amplification product of about 347 bp was then analyzed by electrophoresis through a 1.0% agarose gel and visualized by ethidium bromide staining. The positive amplicons were directly sequenced. Negative and positive control fecal suspensions were included in each assay. RNA extraction and other pre-PCR amplification steps were performed in a separate clean room to minimize cross-contamination.

## Results

### Clinical Signs

Clinical signs, such as diarrhea and jaundice, were not observed; however, some rabbits decreased food consumption for part of the duration of the study. Some rabbits suffered accidental death during blood collection by heart puncture.

### Seroconversion to HEV Antibodies

**Figure 1:**
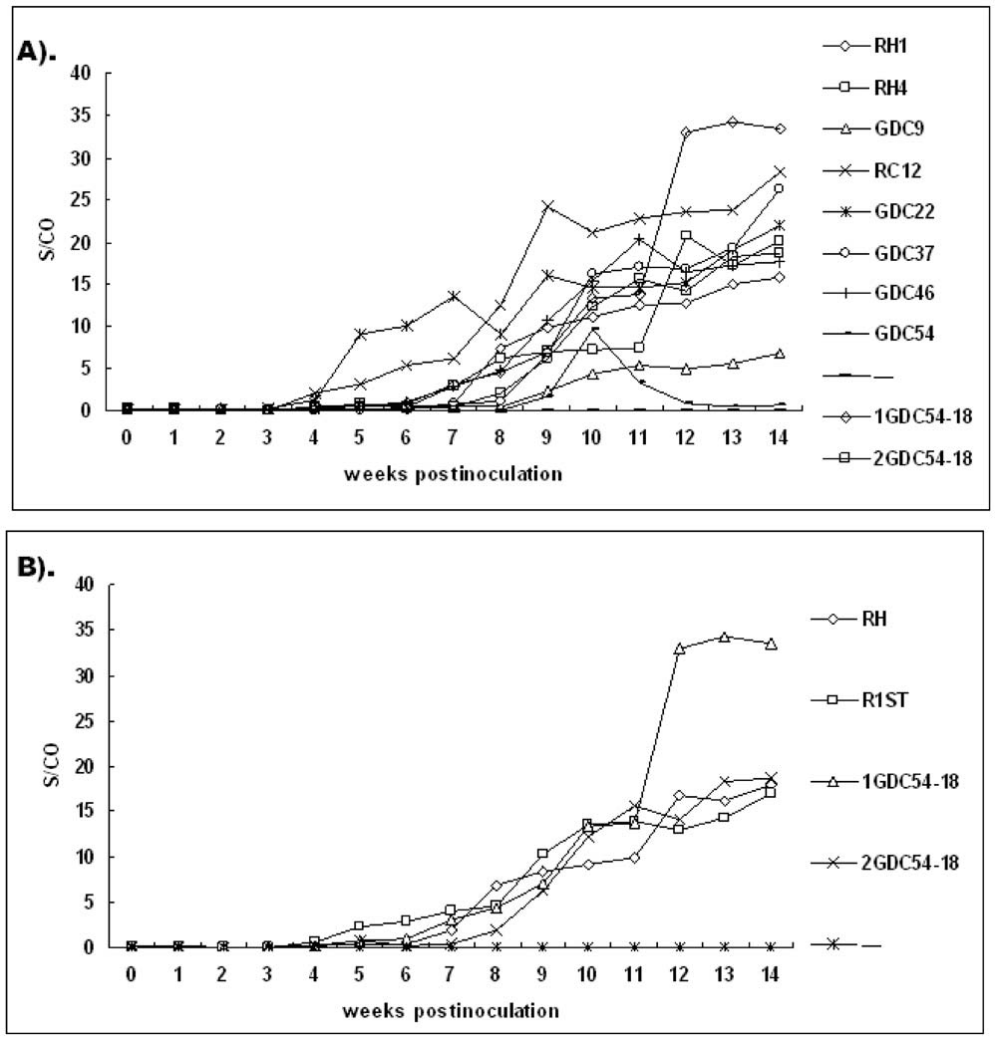
Time course of seroconversion to anti-HEV in SPF rabbits inoculated with different virus isolates. (A) The mean ELISA signal-to-cutoff (S/CO) values for all rabbits from each group at each week post-inoculation are plotted. (B) All rabbits from groups 3 to 8 inoculated with the non-passaged HEV are named R1ST and the combination of groups RH1 and RH4 are named RH.

Prior to inoculation, all rabbits were seronegative for HEV. All control rabbits remained seronegative throughout the study. Anti-HEV IgG was detected in six of nine rabbits inoculated with the RH1 strain HEV (group RH1) and in eight of nine rabbits inoculated with the RH4 strain (group RH4) by 14 weeks post-inoculation (wpi) (Table 2). All rabbits inoculated with rabbit HEV were seropositive at the end of the study with the exception of one rabbit in group GDC54, based on the time point at the end of the study. These rabbits which died before seroconversion were not included. This rabbit was inoculated with GDC54 and was antibody-positive for only 3 weeks but then tested antibody-negative at 12 wpi. The dynamic patterns of anti-HEV IgG antibodies detected in the groups RH1,RH4 and all six groups inoculated with the non-passaged rabbit HEV strains, regardless of whether they were inoculated with genotype 1, 4 or rabbit HEV were very similar in S/CO values that increased gradually and were not statistically different (Fig. 1B). In fact, the changing trends of antibody levels in the inoculated groups were also similar and increased gradually by the end of the study, with the only discernable difference being the time to seroconversion. However, the only groups to not follow these trends were groupGDC54, whose antibody levels peaked at 10 wpi and then returned to the baseline levels at 12 wpi, and group 1GDC54-18, whose antibody levels increased rapidly at 11 wpi (Fig. 1A).

**Table 2:** Time course for seroconversion of rabbits inoculated with HEV.

Inoculum strain (Groups)	No. of seropositive rabbits/total no. tested at indicated weeks post-inoculation														
	0	1	2	3	4	5	6	7	8	9	10	11	12	13	14
RH1	0/9	0/9	0/9	0/9	0/9	0/9	1/9	2/9	5/9	5/9	5/9	6/9	6/9	6/9	6/9
RH4	0/9	0/9	0/9	0/9	0/9	1/9	2/9	4/9	4/8	4/8	4/8	4/8	7/8	7/8	7/8
GDC9	0/2	0/2	0/2	0/2	0/2	1/2	1/2	0/2	0/2	1/1	1/1	1/1	1/1	1/1	1/1
RC12	0/2	0/2	0/2	0/2	1/2	2/2	2/2	2/2	2/2	2/2	2/2	2/2	2/2	2/2	2/2
GDC22	0/2	0/2	0/2	0/2	1/2	1/2	1/2	1/2	1/2	2/2	2/2	2/2	2/2	2/2	2/2
GDC37	0/2	0/2	0/2	0/2	0/2	0/2	0/2	1/2	1/2	1/2	1/2	1/2	2/2	2/2	2/2
GDC46	0/2	0/2	0/2	0/2	0/2	0/2	1/2	1/2	1/2	1/2	1/2	2/2	2/2	2/2	2/2
GDC54	0/2	0/2	0/1	0/1	0/1	0/1	0/1	0/1	0/1	1/1	1/1	1/1	0/1	0/1	0/1
Control	0/4	0/4	0/4	0/4	0/4	0/4	0/4	0/4	0/4	0/4	0/4	0/4	0/4	0/4	0/4
1GDC54-18	0/4	0/4	0/4	0/4	0/4	0/3	1/3	1/3	2/3	1/3	2/3	2/3	1/1	1/1	1/1
2GDC54-18	0/4	0/4	0/4	0/4	0/4	0/4	0/4	1/4	1/4	4/4	4/4	4/4	4/4	4/4	4/4

### Detection of HEV Antigens

**Figure 2:**
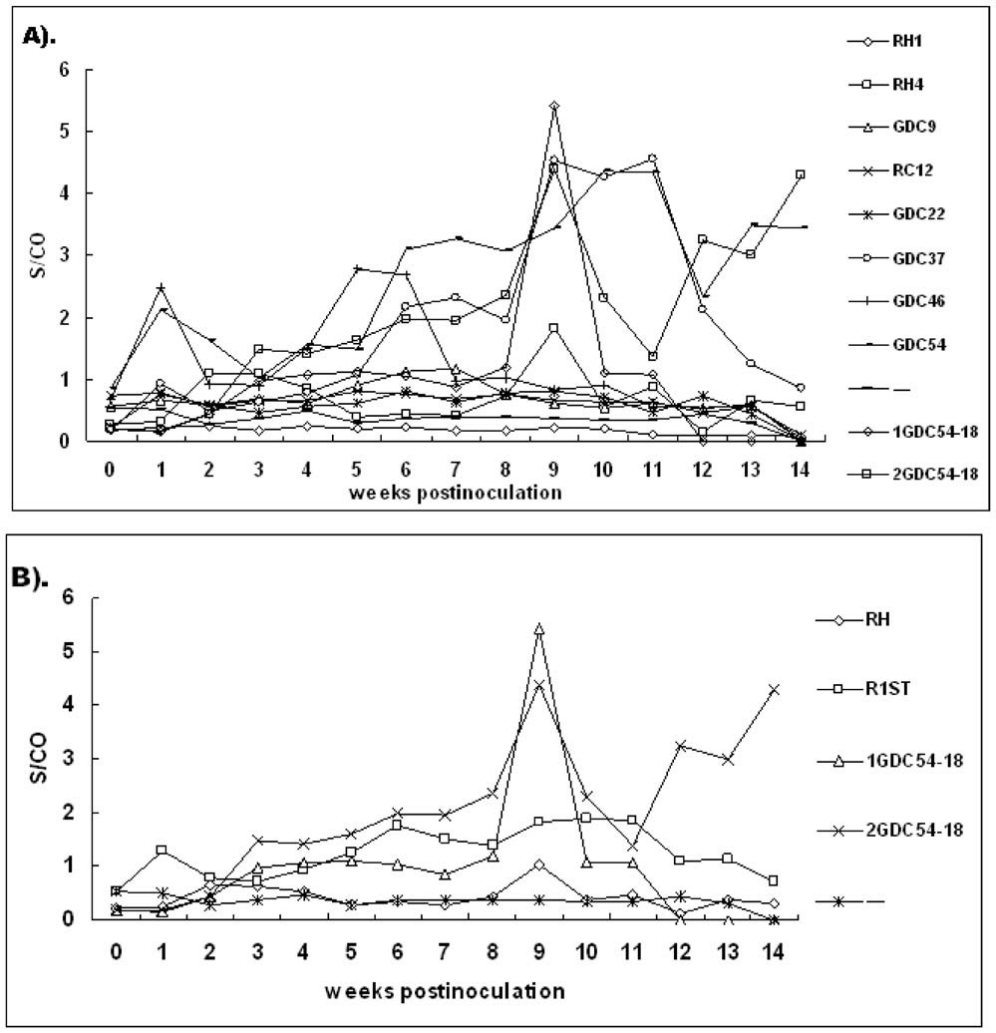
Time course of serological appearance of HEV antigens in inoculated SPF rabbits. (A) The weekly mean ELISA signal-to-cutoff (S/CO) values for all rabbits from each group are plotted. (B) All rabbits from groups 3 to 8 inoculated with the non-passaged HEV are named R1ST and the combination of groups RH1 and RH4 are named RH.

All rabbits were negative for HEV antigen prior to inoculation and no control rabbits seroconverted throughout the study. The mean S/CO values of antigenemia differed between the rabbits inoculated with genotypes 1 and 4 and rabbits inoculated with the non-passaged rabbit HEV isolates (p<0.0001) (Fig. 2B). HEV antigen was detected in the sera of two of the nine group RH4 rabbits, one from 2 to 4 wpi and the other from 6 to 14 wpi. No antigen was detected in group RH1 rabbits during the course of observation (Table 3). HEV antigen was detected in half of rabbits inoculated with the non-passaged rabbit HEV isolates, the main differences being in the time to peak antigen levels (Fig. 2A). Some rabbits inoculated with GDC54 showed double peaks, regardless of whether original isolates or second-passage inocula were used. The antigen levels in the 2GDC54-18 inoculated group of rabbits remained elevated from 1 to 9 wpi and from 11 to 14 wpi, until the end of the study. For group 1GDC54-18, antigen levels also showed a peak at 9 wpi and returned to baseline levels at 12 wpi. It is noteworthy that just one rabbit of this group survived until the end of the study because of the accidental death of the others at the 11 wpi. This surviving rabbit was negative for HEV antigen throughout the study.

**Table 3:** Time course for the presence of HEV antigens in the sera of inoculated rabbits.

Inoculum (Groups)	No. of seropositive rabbits/total no. tested at indicated weeks post-inoculation														
	0	1	2	3	4	5	6	7	8	9	10	11	12	13	14
RH1	0/9	0/9	0/9	0/9	0/9	0/9	0/9	0/9	0/9	0/9	0/9	0/9	0/9	0/9	0/9
RH4	0/9	0/9	1/9	1/9	1/9	0/9	1/9	1/9	1/8	1/8	1/8	1/8	0/8	1/8	1/8
GDC9	0/2	0/2	0/2	0/2	0/2	1/2	1/2	1/2	0/2	0/1	0/1	0/1	0/1	0/1	0/1
RC12	0/2	0/2	0/2	0/2	0/2	0/2	0/2	0/2	0/2	0/2	0/2	0/2	0/2	0/2	0/2
GDC22	0/2	0/2	0/2	0/2	0/2	0/2	0/2	0/2	0/2	0/2	0/2	0/2	0/2	0/2	0/2
GDC37	0/2	0/2	0/2	0/2	0/2	1/2	1/2	1/2	1/2	1/2	1/2	1/2	1/2	1/2	1/2
GDC46	0/2	1/2	1/2	1/2	1/2	1/2	1/2	1/2	1/2	1/2	1/2	0/2	0/2	0/2	0/2
GDC54	0/2	2/2	1/1	1/1	1/1	1/1	1/1	1/1	1/1	1/1	1/1	1/1	1/1	1/1	1/1
Control	0/4	0/4	0/4	0/4	0/4	0/4	0/4	0/4	0/4	0/4	0/4	0/4	0/4	0/4	0/4
1GDC54-18	0/4	0/4	1/4	1/4	1/4	1/3	1/3	1/3	1/3	2/3	1/3	1/3	0/1	0/1	0/1
2GDC54-18	0/4	0/4	0/4	3/4	3/4	2/4	2/4	2/4	2/4	2/4	2/4	2/4	2/4	2/4	2/4

### Detection of HEV RNA in Serum and Fecal Samples from Rabbits

Pre-inoculation samples taken from all rabbits were negative for HEV RNA and all uninoculated control rabbits remained negative throughout the experiment. Rabbit HEV RNA was detected variably in serum and fecal samples from rabbits in the various groups (Table 4).

**Table 4:** Detection of HEV RNA in sera and feces collected weekly from rabbits inoculated with HEV isolates.

Inoculum (Groups)	No. of positive sera (no. of positive feces)/total no. tested at indicated weeks post-inoculation														
	0	1	2	3	4	5	6	7	8	9	10	11	12	13	14
RH1	0(0)/9	0(0)/9	0(0)/9	0(0)/9	0(0)/9	0(0)/9	0(0)/9	0(0)/9	0(0)/9	0(0)/9	0(0)/9	0(0)/9	0(0)/9	0(0)/9	0(0)/9
RH4	0(0)/9	2(0)/9	2(2)/9	2(1)/9	1(1)/9	0(0)/9	1(1)/9	0(1)/9	1(1)/8	0(0)/8	1(0)/8	1(1)/8	0(1)/8	1(0)/8	0(0)/8
GDC9	0(0)/2	0(0)/2	0(1)/2	0(0)/2	0(1)/2	0(2)/2	0(1)/2	0(1)/2	1(1)/2	0(1)/1	0(0)/1	0(0)/1	0(0)/1	0(0)/1	0(0)/1
RC12	0(0)/2	0(0)/2	0(0)/2	0(0)/2	0(0)/2	0(0)/2	0(0)/2	0(0)/2	0(0)/2	0(0)/2	1(0)/2	0(0)/2	0(0)/2	0(0)/2	0(0)/2
GDC22	0(0)/2	0(0)/2	0(0)/2	0(0)/2	0(0)/2	0(1)/2	0(0)/2	0(1)/2	0(1)/2	0(1)/2	0(1)/2	0(1)/2	0(1)/2	0(1)/2	0(1)/2
GDC37	0(0)/2	0(0)/2	0(0)/2	0(2)/2	0(2)/2	0(1)/2	0(1)/2	0(1)/2	0(1)/2	0(1)/2	0(1)/2	0(1)/2	0(1)/2	0(1)/2	0(1)/2
GDC46	0(0)/2	0(0)/2	0(0)/2	0(0)/2	0(1)/2	0(1)/2	0(1)/2	0(1)/2	0(1)/2	0(1)/2	1(2)/2	0(0)/2	0(0)/2	0(0)/2	0(0)/2
GDC54	0(0)/2	0(0)/2	0(0)/1	0(1)/1	0(1)/1	0(1)/1	0(1)/1	0(1)/1	0(1)/1	0(1)/1	0(1)/1	0(1)/1	0(1)/1	0(1)/1	0(1)/1
Control	0(0)/4	0(0)/4	0(0)/4	0(0)/4	0(0)/4	0(0)/4	0(0)/4	0(0)/4	0(0)/4	0(0)/4	0(0)/4	0(0)/4	0(0)/4	0(0)/4	0(0)/4
1GDC54-18	0(0)/4	0(1)/4	1(0)/4	2(0)/4	0(1)/4	1(1)/3	1(2)/3	0(1)/3	0(1)/3	0(1)/3	0(1)/3	0(1)/3	0(0)/1	0(0)/1	0(0)/1
2GDC54-18	0(0)/4	2(1)/4	1(0)/4	2(2)/4	2(3)/4	0(1)/4	1(3)/4	2(3)/4	2(3)/4	2(1)/4	1(1)/4	1(1)/4	0(1)/4	0(1)/4	1(2)/4

Viremia was detected in sera from 1 to 3 wpi in two of nine RH4 group rabbits inoculated with genotype 4 HEV, with one animal intermittently positive thereafter. No viral RNA was detected in any rabbit of the RH1 group inoculated with HEV genotype 1. Viremia was detected only three times among any of the six groups of rabbits inoculated with the non-passaged rabbit HEV isolates, including one of two rabbits in the GDC9 group at 8 wpi, one of two in the RC12 group at 10 wpi and one of two in the GDC46 group at 10 wpi. For the 1GDC54-18 group rabbits, viremia was detected from 1 to 2 wpi and 5 to 6 wpi. HEV RNA was first detected in the serum of two of four rabbits in the 2GDC54-18 group at 1 wpi and was intermittently positive thereafter, up to 11 wpi.

Fecal shedding of viruses was not detected in group RH1. For group RH4 rabbits, HEV RNA was first detected in feces from two of the nine rabbits at 2 wpi and one them was intermittently positive thereafter. All of the groups inoculated with the non-passaged rabbit HEV isolates were intermittently positive for HEV RNA in feces, except group RC12. For the group 1GDC54-18 rabbits, HEV RNA was shed in the feces first at 1 wpi and lasted until 4 to 11 wpi. Three of the four group 2GDC54-18 rabbits were HEV RNA positive at 4 wpi and from 6 to 8 wpi.

The viruses recovered from selected experimentally-infected rabbits were sequenced to confirm that they were derived from the original inoculum, with nucleotide identity 99–100% to the original inoculum.

### ALT Levels in Serum

**Figure 3:**
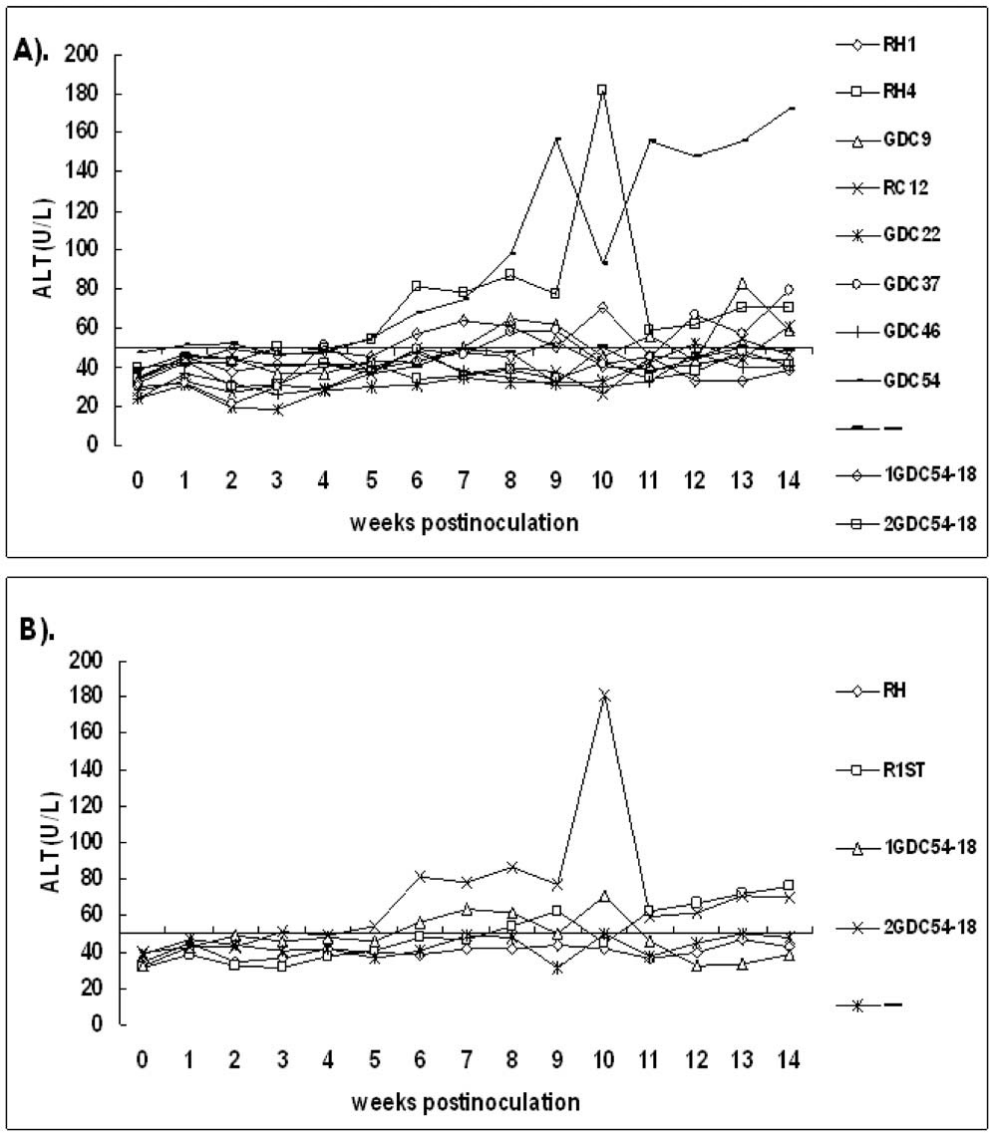
Levels of the lalanine aminotransferase (ALT) in sera from inoculated and control rabbits. (A) The mean ALT values of all rabbits from each group at each week post-inoculation are plotted. (B) All rabbits from groups 3 to 8 inoculated with the non-passaged rabbit HEV are named R1ST and the combination of groups RH1 and RH4 are named RH.

No instant elevations in serum ALT-levels were observed in the four control rabbits, nine rabbits each in groups RH1 and RH4 during the entire study (Fig. 3A). However, an increasing trend was observed for each rabbit, and significantly increased serum ALT levels were observed during the latter period of fecal HEV RNA excretion for two RH4 group rabbits. The differences in minimum and maximum values of serum ALT for these two rabbits were 63 and 97 U/L. For the 12 rabbits inoculated with the first-generation rabbit HEV, the ALT responses differed over time, similar to the occurrence of antigens and HEV RNA. For the RC12 and GDC46 group rabbits, levels of ALT in sera showed no obvious increase during the study. ALT levels of GDC54 group rabbits were significantly elevated, with a peak value of 157 U/L at 9 wpi and 172 U/L at 14 wpi. Therefore, we selected viruses recovered from this infected group for the second-generation infectious stocks. Accordingly, dramatic elevations in serum ALT were observed for two 2GDC54-18 group rabbits with peak values at 10 wpi as high as 181 and 426 U/L, respectively. The 1GDC54-18 group, however, was an exception as peak ALT levels were not high, probably due to the frequent occurrence of accidental death not associated with HEV infection. The strongest ALT response was observed in the 2GDC54-18 group (Fig. 3B). Overall, the rabbits in six groups inoculated with non-passaged rabbit HEV isolates showed a stronger ALT response than rabbits inoculated with genotypes 1 and 4 despite those rabbits being inoculated with much higher doses in terms of genome equivalents of viruses.

### Liver Histopathology

**Figure 4:**
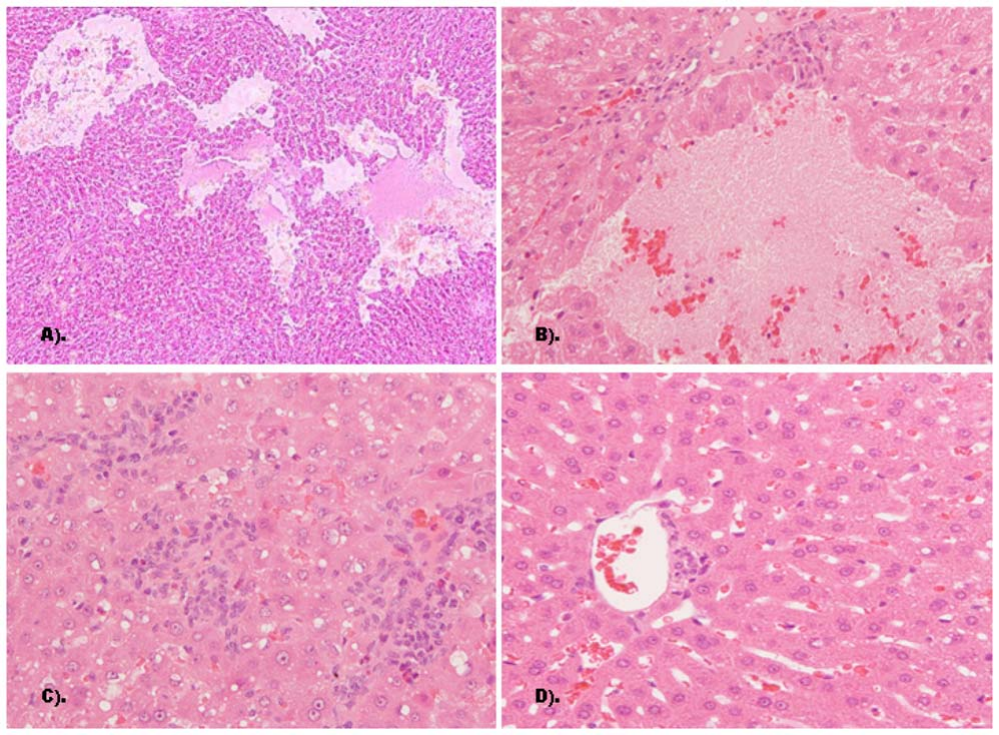
Pathological signs of HEV infection in hematoxylin and eosin stained liver sections. (A & B) Liver sections from a group 2GDC54-18 rabbit showing localized extensive hepatocellular necrosis (magnification 10× and 20×, respectively). (C) Liver section from a group RH4 rabbit showing irregularly distributed multifocal lymphohistiocytic infiltrates (20×). (D) Liver section from a control rabbit showing no visible pathological signs of HEV infection (20×).

No statistical data on the pathological signs of HEV infection in the liver can be reported because liver biopsies were not performed and no rabbits were regularly necropsied during the entire study. Some rabbits that showed elevated ALT levels when they were necropsied at the end of the study or died due accidentally and all control rabbits were investigated for liver pathology. Multifocal lymphohistiocytic infiltrates were distributed irregularly in the liver of a rabbit from group RH4 (Fig. 4C) and locally hepatocellular necrosis was observed in liver sections from a 2GDC54-18 group rabbit (Fig. 4A and B). No pathological signs of HEV infection were observed in liver sections of control group rabbits (Fig. 4D).

## Discussion

The lack of an effective cell culture system and a small animal model has hampered the progress of HEV research. Experimental infections of pigs and non-human primates with HEV have been well documented [33], [13], [16], [23], [24], [34], [20], [19]. In addition, chickens [26] and rats [35] have also been assessed as animal models for studying HEV replication and pathogenesis. The discovery of a novel genotype of HEV that infects rabbits prompted us to evaluate these animals as a model for the study of HEV. Full-length sequence and phylogenetic analyses have revealed that the sequences isolated from rabbits represent a novel genotype of HEV genetically related to mammalian HEV, most closely to genotype 3. In addition, test data showed that there is significant cross-reactivity of antibodies produced during infection of both rabbit HEV and HEV genotypes 1 and 4, especially to the ORF2 polypeptides (data not shown). The current study investigated rabbit HEV replication and pathogenesis in a homologous animal model and also determined whether HEV genotypes 1 and 4, isolated from humans, also can infect rabbits.

Six original isolates of rabbit HEV were used to infect 12 SPF rabbits. Based on a 304 bp region of ORF2, the rabbit HEV strains used in this study shared 84–99% identity and are 73–77, 70–76, 75–82, 71–77, and 53–65% identical to the corresponding regions of human HEV genotypes 1, 2, 3, 4, and avian HEV, respectively [10]. The maximum dose of virus available for each strain was used to infect rabbits. The markers of infection, HEV RNA and antigen, and of seroconversion, anti-HEV antibody, were detected in sera or feces of inoculated and non-inoculated rabbits. The sensitivity for RT-PCR using in the present study can reach to 5.6×10^3^ copies/reaction [36] to detect HEV RNA. The concordances between HEV antigen and HEV RNA detected by using the same assays as in the present study was 77.1% with significant correlations [37]. Consequently, the clinical symptoms manifested among the different groups varied. Overall, all rabbits inoculated with the non-passaged rabbit HEV isolates seroconverted to HEV by 3 months postinoculation, except for animals that died accidentally midway through the study. The timing of the appearance of antibody was related to other clinical markers of disease, i.e., if viral antigens appeared early in sera, then the corresponding antibodies appeared later. As expected, rabbits inoculated with lower doses of virus in terms of genome equivalents showed a weaker serological response. For example, rabbits inoculated with RC12 strain of HEV (4.69×10^1^ genome equivalents) seroconverted at 4 wpi without antigenemia or HEV RNA in feces. In contrast, rabbits in the GDC54 inoculated group developed more severe clinical symptoms following challenge with a larger virus inoculum (6.93×10^4^ genome equivalents). Nonetheless, we infected rabbits successfully with HEV isolated from rabbits and proved that they are a suitable animal model for the study of rabbit HEV.

Because the virulence of the first-generation rabbit HEV was limited, we used virus recovered from the feces of rabbits inoculated with the GDC54 strain of HEV as a second passage inoculum for the further studies. The second passage HEV GDC54-18 virus inoculum was used to infect another eight rabbits, using two different doses. As expected, challenging with higher doses of virus in terms of genome equivalents caused a more severe hepatitis. Serum ALT levels, which are indicative of recent liver damage and suggestive of an acute infection, peaked at 10 wpi (Fig. 3B) with a value up to 181 U/L for 2GDC54-18 group rabbits. In addition, local hepatocellular necrosis was observed in liver sections from a rabbit in the 2GDC54-18 group (Fig. 4A and B). Unfortunately, only one of four 1GDC54-18 group rabbits survived up to 12 wpi because of the accidental death of the others in the group, not associated with virus infection. The results from this study indicate that rabbits are a useful animal model for HEV infection due to the recapitulation of hepatic disease and typical HEV replication and pathogenesis. However, only few rabbits were necropsied at non-planned time points, a larger number of animals with higher infectious dose and planned optimal necropsy schedule are needed to definitively characterize the hepatic lesions associated with rabbit HEV infection in rabbits. Such a study will be designed in future.

Another objective of this study was to determine whether human HEV could infect rabbits. So far, no evidence has proved that human genotypes 1 and 2 HEV can infect pigs, although genotypes 3 and 4 can infect both human and pigs. In addition to pigs, many other animals have been shown to be susceptible to infection with genotypes 3 and 4 HEV and can serve as a reservoir of HEV that can amplify the virus and transmit to humans [38], [39], [40], [41], [42], [43], [12], [26], [10]. Although different HEV types have very similar genomic organization, they may vary in their ability to infect different mammalian species. Whether rabbit HEV may be transmitted to humans is not known [10]. In this study, No HEV RNA and antigens were observed in the sera of the nine RH1 group rabbits, with no fecal shedding. Serum ALT levels showed no instant elevations in accordance with the lack of pathological signs of HEV infection in liver. However, six of nine animals became anti-HEV antibody positive. The doses of virus, in terms of genome equivalents, inoculated into the animals were much higher for this group than for groups inoculated with non-passaged rabbit HEV isolates. Whether the antibody was induced by HEV infection or immunization with a high dose HEV is not clear and requires further study. For the RH4 group rabbits inoculated with the highest virus inoculum (1.14×10^7^ genome equivalents) of all test animals, seven rabbits seroconverted at 14 wpi. In summary, seroconversion, viremia, fecal virus shedding or serum liver enzyme elevation were observed in two of nine RH4 group rabbits, suggesting evidence of virus infection and hepatitis. The results indicate that HEV genotype 4 have the ability to cross species and infect rabbits. As we have reported previously, genotype 1 and 4 HEV can infect monkeys and all animals were infected and developed severe hepatitis [30]. It is noteworthy that the challenge doses (the ratio of genome equivalents of virus to the weight of animal) were similar in the rabbit and monkey models. Therefore, the infectivity and pathogenicity of genotype 4 HEV in rabbits was much lower than seen in non-human primate models of infection.

In summary, we infected rabbits successfully with rabbit HEV and the clinical picture in these animals was similar to HEV infection of monkeys. It seems that rabbits constitute a valuable model to study the mechanisms of HEV replication and pathogenesis. These studies indicate the rabbit model has potential for the development of HEV vaccines and clinical therapies for the treatment of infection.
